# Osteopoikilosis: A rare cause of bone pain 

**Published:** 2015

**Authors:** Jgirim Mahbouba, Golli Mondher, Mhenni Amira, Manari Walid, Bergaoui Naceur

**Affiliations:** 1 Department of Rheumatology of Monastir, University of Medicine of Monastir, Monastir, Tunisia.; 2 Department of Radiology of Monastir, University of Medicine of Monastir, Monastir, Tunisia.

**Keywords:** Osteopoikilosis, Diagnosis, Osteoblastic Metastasis, Treatment.

## Abstract

**Background:**

Osteopoikilosis (OPK) is a rare inherited condition of the bones, transmitted as an autosomal dominant trait characterized by numerous hyperostotic areas that tend to localize in periarticular osseous regions. It is usually asymptomatic and is often diagnosed incidentally during x-rays made by other reasons. We present a case of 34-year-old man suffering from polyarthralgia and low back pain.

**Case presentation:**

A 34-year-old male patient, smoking 40 packs yearly and alcoholic was referred to our department of rheumatology, complaining of polyarthralgia which started 3 years ago and involving large and small joints. He reported the presence of pelvic pain mostly located at both hip joints and in the two ankles. On radiologic examination, numerous, symmetric, well defined, sclerotic lesions were identified on shoulder, wrist, ankles, pelvis, and on spine. The size of the lesions varied from 2 to 9 millimeters. These spots were located on spongious bone tissue, and in the inner bone cortex located bilaterally in the epiphyses and metaphyses. We concluded the diagnosis of OPK. His mother was found to have the same lesions without any symptoms.

**Conclusion:**

OPK may be an isolated finding or associated with other pathologies, e.g. skin manifestations, rheumatic and/or skeletal disorders. The main differential diagnosis is osteoblastic metastasis.

**O**steopoikilosisis is a sclerosing bony dysplasia of unknown etiology with multiple enostosis. It is a rare inherited benign condition incidentally found on squeletal x rays. It is characterized by an abnormality in bone maturation process and often found incidentally on radiologic examination. An autosomal dominant inheritance has been proposed for OPK. Albers-Schonberg was the first to describe this uncommon sclerosing bone dysplasia in 1915 (1). Incidence in both sexes is identical and it can occur at any age. Pain is not a prominent feature of OPK, but in some patients, pain could be a presenting symptom of the disorder. 

The signals of this rare hereditary condition are generally found incidentally on plain radiographs. Sometimes this disorder is associated with other abnormalities such as dacryocystitis, dermatofibrosis lenticularis disseminate, scleroderma, discoid lupus erythematosus, keloids, syndactyly, cleft palate, heart or renal malformations, endocrine disorders, and autoimmune disorders (2-4). The principal considerations in differential diagnosis in the cases of OPK are mastocytosis, tuberous sclerosis, and mainly osteoblastic metastasis. This case shows the importance to keep OPK in mind in patients with diffuse pain to avoid misdiagnosis and invasive diagnostic procedures.

## Case Report

A 34-year-old-male patient, smoking 40 packs yearly and alcoholic was referred to our department of rheumatology, complaining of polyarthralgia which started 3 years ago and involving large and small joints. He reported the presence of pelvic pain mostly located at both hip joints and in the two ankles. His pain was constant, but worsened in long distance walking. The quality of pain was vague and its severity was rated as 4 on the visual analog scale. He did not have any systemic disease and was not taking any medication. There was no history of trauma, morning stiffness, weight loss, fever and pain; nor any sign of arthritis. He reported a history of past trauma causing a fracture on the right wrist. There was no visible gait abnormality or postural deviations. Inspection of pelvis showed bilateral symmetry of muscle bulk and there were no scars or bruises. 

**Figure 1:**
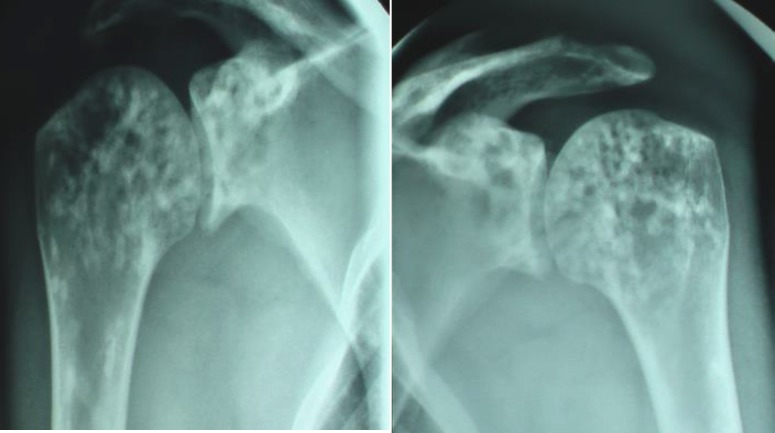
Anteroposterior radiograph of the shoulders demonstrating multiple sclerotic foci in the proximal humerus and scapula adjacent to the glenohumeral joint

**Figure 2:**
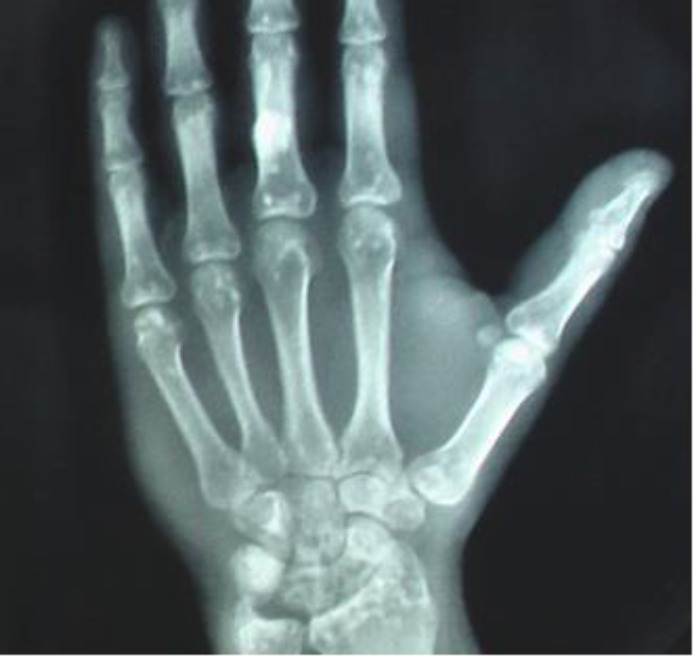
Osteopoikilotic lesions on the hand

On physical exam, the range of motion of all joints was normal with no restriction. Musculoskeletal examination has not objectified synovitis except a swelling at the right Achilles tendon. Neurologic examination revealed no abnormality. Sacroiliac joint tests were also normal. No sign of cutaneous connective tissue involvement noted. The laboratory, all findings for complete blood count, erythrocyte sedimentation rate, C-reactive protein, rheumatoid factor, serum electrolytes, alkaline phosphatase, calcium, phosphate and thyroid function tests were normal. On radiologic examination, numerous, symmetric, well defined, sclerotic lesions were identified on the shoulder (figure 1), wrist (figure 2), ankles, pelvis, and on the spine. 

**Figure 3:**
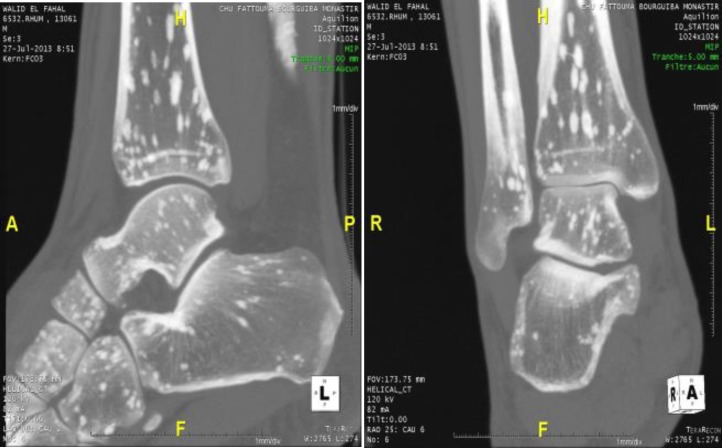
Computed tomography of the ankle: anteroposterior and lateral radiographs of the ankle. Sclerotic foci of variable size appear in the tibia and calcaneum

The size of the lesions varied from 2 to 9 millimeters. These spots are located on spongious bone tissue, and in the inner bone cortex located bilaterally in the epiphyses and metaphyses. Computed tomography scan of the pelvis and lumbar spine has not objectified lumbar spinal stenosis. Computed tomography of the ankles has objective sclerotic foci in the tibia and calcaneum of variable size (Figure 3). 

With these clinical and radiologic findings, we concluded the diagnosis of OPK. Management with nonsteroidal anti-inflammatory drugs (NSAIDs) and opioid analgesics resulted in improved pain managment. Currently, he continues to require pain medications to maintain his daily activities. Because of the familial nature of the disease, his family members were also evaluated. His mother was found to have the same lesions without any symptoms while his father, his brother and his sister were lesionfree.

## Discussion

OPK, also called osteopathia condensans disseminata, asymptomaic bone dysplasia, a spotted bone disease, is a rare disease. It can be transmitted as autosomal dominant or can be sporadic (5). The overall incidence of OPK has been claimed to be one in every 50,000 subjects (2). Lesions are seen in the metaphysis and epiphysis of long bones. Lesions in the dorsolumbar spine were reported in a patient by Weisz (6). Typically the patients are asymptomatic, although as many as 20 % may have mild articular pain and joint effusion (7). The main complaint in our patient which caused him to seek medical help was persistent pain in affected areas. Some hypotheses have been made to explain the mechanisms of pain in OPK. It is presumed that joint pain is an incidental finding in the course of OPK. Increased localized bone metabolism at the location of the lesion, irritation of joint capsule attachment by sclerotic areas and increased intraosseous pressure due to venous stasis at the areas of lesion can produce joint pain. Patient examination was not remarkable and no deformity or dysfunction could be documented. Fracture is a rare presentation of the disease (8). Our patient had a fracture of the fifth metacarpal. Spinal stenosis has been reported in patients with OPK (6). CT scan of the pelvis and lumbar spine of our patient has not objectified lumbar spinal stenosis but it shows multiple enostosis located on the vertebrae. 

Radiologic signs of OPK are similar to our finding. The bone islands typically clustered around the joints and align themselves parallel to surrounding trabeculae (thus predominantly longitudinally in the metaphyses). The commonly involved sites are metaphysis and epiphysis of long bones, scapulae, pelvis, carpi and tarsi (2). OPK is normally symmetric, numerous, varies in size from a few millimeters to several centimeters, well-defined, homogeneous, and circular or ovoid. Differential diagnosis in plain radiographs may be with mastocytosis, tuberous sclerosis and, principally, osteoblastic metastasis (9). MRI abnormalities observed in our case are the same as those described in the literature. Each lesion is small and dark on both T1 and T2 weighted images, as it is composed of mature dense bone.

Bone scan findings are usually normal in patients with OPK as they were found in our patient, but reveal slightly increased activity similar to the bone island or enostosis that reflects active osseous remodeling. There is no consensus on literature about the treatment. Non-steroidal anti-inflammatory drugs (NSAIDs) are often used as an option for the treatment of pain. Analgesics such as acetaminophen and opioids can also be used. Rare active lesions have been treated with bisphosphonate therapy, but the results are controversial (10). Our patient was treated with NSAIDs, and opioid analgesics for pain management. 

Differential diagnosis from osteoblastic metastasis must be done before performing invasive diagnostic particularly in symptomatic patients and to prevent false alarm for the patients. **OPK** is one of the skeletal “don’t touch” lesions. Thus, well-timed follow-up visits of the patients are recommended to survey other conditions which may require treatment.
